# Nasal Manifestations of Immunoglobulin A (IgA) Vasculitis in an Adolescent Patient: A Case Report

**DOI:** 10.7759/cureus.110592

**Published:** 2026-06-10

**Authors:** Ifigeneia Ganna, Antonia Arvaniti, Irini Kalogera, Nektarios Papapetropoulos, Theodoros Pantazopoulos

**Affiliations:** 1 Otolaryngology, Agia Sofia Children's Hospital, Athens, GRC; 2 Otolaryngology, University of Crete, Heraklion, GRC

**Keywords:** chronic rhinitis, iga vasculitis (igav), iga vasculitis nephritis, nasal biopsy, renal biopsy

## Abstract

Immunoglobulin A vasculitis (IgAV), formerly known as Henoch-Schönlein purpura, is a complex immune-mediated vasculitis characterized by the involvement of small blood vessels in various organ systems.

We describe the case of a 14-year-old girl who developed a non-typical manifestation of vasculitis with notable rhinological features. The patient presented with a vasculitis-type rash on the hands and lower extremities bilaterally and arthralgia, while she also complained about intense nasal congestion. The laboratory results were atypical for a specific disorder, and the skin and nasal mucosa biopsies were not diagnostic. Since the patient presented with microscopic hematuria, she underwent a kidney biopsy that confirmed the diagnosis of IgAV with nephritis. The patient was maintained on long-term therapy with corticosteroids, azathioprine, and hydroxychloroquine and showed improvement in symptoms.

The aim of this case report is to highlight the atypical presentation of IgAV, particularly its rhinologic manifestations, which are infrequently reported in the literature. In addition, this report emphasizes the importance of long-term follow-up in such patients, as the diagnosis can be challenging.

## Introduction

Vasculitis comprises a heterogeneous group of disorders characterized by inflammation and necrosis of blood vessel walls, often resulting in tissue ischemia and organ dysfunction [1, 2]. IgA vasculitis (IgAV) is an immunoglobulin A (IgA)-mediated vasculitis characterized by IgA1-dominant immune deposition in the affected vessel walls. IgAV primarily affects the small vessels of the joints, kidneys, gastrointestinal tract, skin, and upper airway mucosa [3]. The diagnosis should be based on the finding of palpable purpura in the presence of at least one of the following criteria: diffuse abdominal pain, arthritis or arthralgia, renal involvement (hematuria and/or proteinuria), and a biopsy showing predominant IgA deposition. The average duration of the disease in children is four weeks; it is usually self-limited, and long-term complications are rare [1].

Involvement of the upper respiratory tract, particularly the nasal cavity and paranasal sinuses, is a known feature of several forms of systemic vasculitis, such as granulomatosis with polyangiitis and eosinophilic granulomatosis with polyangiitis [4, 5], but little is known about the association between IgAV and chronic rhinitis or rhinosinusitis. Nasal and sinus manifestations, including nasal congestion, mucus discharge, crusting and ulceration of the nasal mucosa, septal perforation, and nose deformity, may appear as early or late manifestations of autoimmune disease [6]. These symptoms are nonspecific and may mimic more common ear, nose, and throat (ENT) conditions, contributing to diagnostic delays. We report an unusual case of IgAV in a 14-year-old girl, in whom nasal manifestations constituted some of the earliest and most prominent clinical findings.

## Case presentation

**Figure 1:**
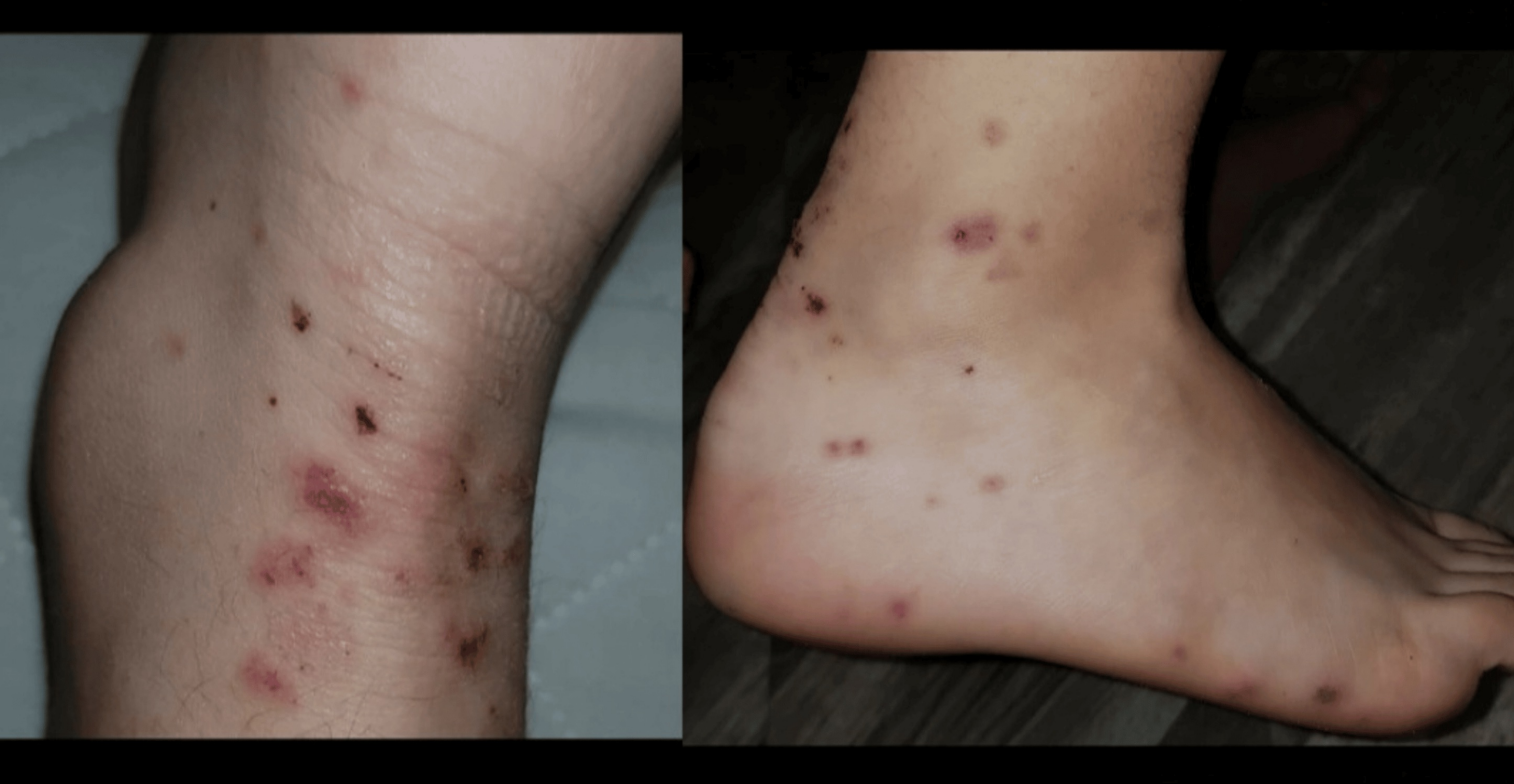
Haemorrhagic rash on lower extremities

**Figure 2:**
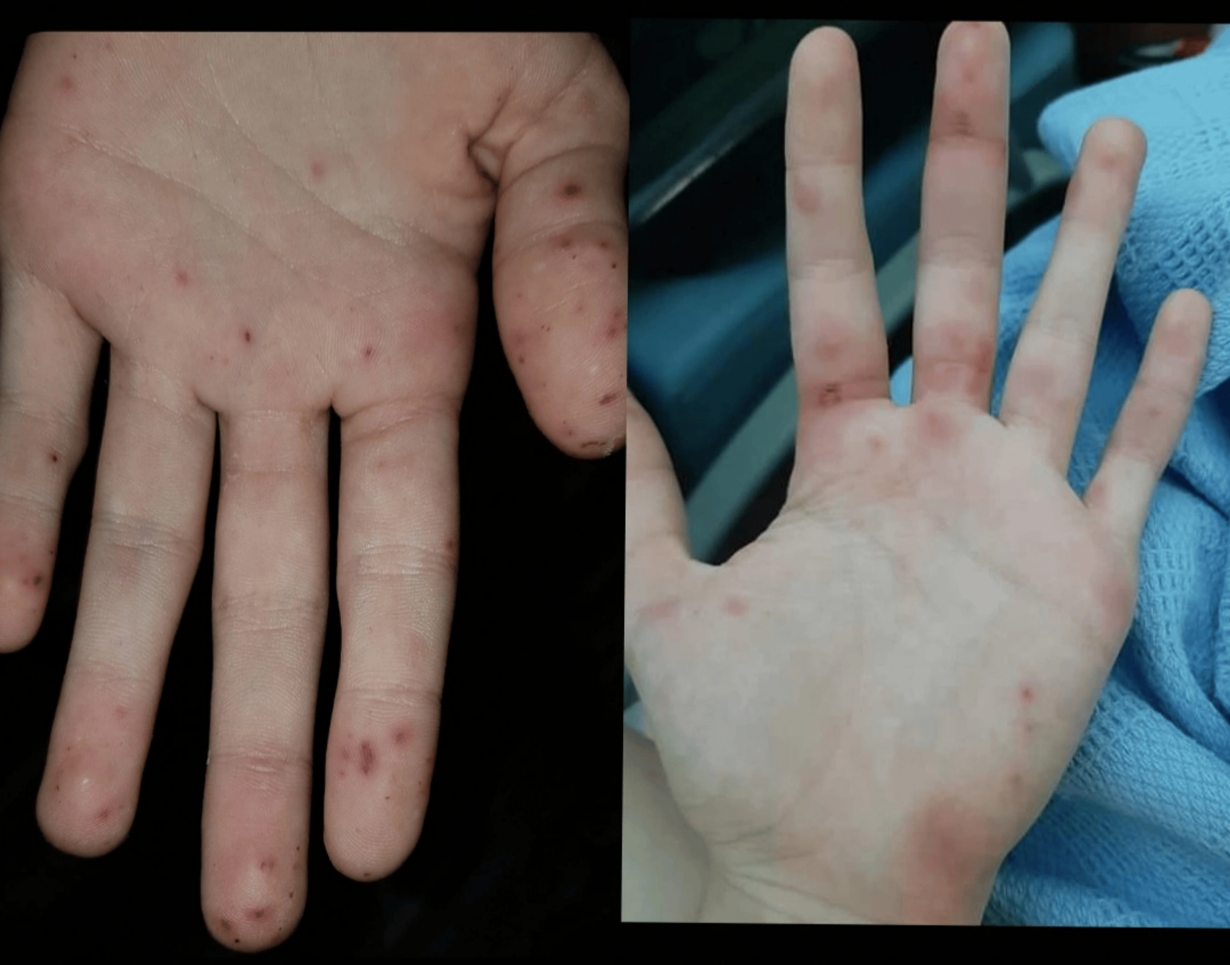
Haemorrhagic rash on hands

A 14-year-old girl was referred by her pediatrician to our tertiary pediatric hospital because of a recurrent vasculitic rash involving both hands and lower extremities (Figures 1, 2), accompanied by arthralgia over the past two months. She also had right calf pain for the preceding five days. The mother stated that the child had not exhibited fever, diarrhea, or macroscopic hematuria, but had been complaining of intense nasal congestion. During the initial episode, two months earlier, the rash had been attributed to a Coxsackie virus infection. Her past medical history indicates Hashimoto thyroiditis, for which she is under treatment with levothyroxine; she also reported taking iron supplementation. She is fully vaccinated, and her perinatal history was unremarkable. Furthermore, no other autoimmune or chronic diseases were reported in the family history.

At the visit, the patient was alert and conscious. Her vital signs were normal for her age. During skin examination, a vasculitis-type rash was observed on both hands and feet as well as the calves. She complained of pain in the right calf and diffuse joint pain without movement impairment. She also had psoriasiform lesions on the elbows. A painful aphthous ulcer was observed in the oral cavity. Chest examination was clear, and the cardiovascular examination was normal. The abdomen was soft on palpation, without pain or organomegaly. The neurologic evaluation revealed no meningeal signs. She was admitted to the pediatric clinic for further pediatric and rheumatologic investigation.

The laboratory evaluation revealed normal values for the full blood count, electrolytes, liver and renal function tests, and C-reactive protein (CRP). The erythrocyte sedimentation rate (ESR) was moderately elevated (ESR value: 35 mm/hr, normal; <10 mm/hr). The immunological assessment was negative except for slight increases in immunoglobulin A (value: 250 mg/dl, normal; 85-214 mg/dl), in immunoglobulin E (value: 395 IU/ml, normal; 11-210 IU/ml) (Table 1), as well as positive atypical antineutrophil cytoplasmic antibodies (atypical ANCA) with a titer of 1:160, while perinuclear ANCA (p-ANCA) and cytoplasmic ANCA (c-ANCA) were negative (Table 2). The urine analysis showed microscopic hematuria (30-35 red blood cells/high-power field) without proteinuria (Table 3). A dermatologic assessment was performed, and a skin biopsy was recommended. An ENT evaluation was also conducted, and nasal endoscopy revealed multiple ulcerative lesions and crusts in the nasal mucosa, specifically on the nasal septum and turbinates. According to the patient, nasal congestion was the most troublesome symptom. The ENT specialist recommended topical application of a nasal moisturizing ointment and daily nasal irrigation with saline solution (sodium chloride, 0.9%).

**Table 1:** Laboratory findings

Immunology and serology factors, ESR	Values	Reference values
Total immunoglobulin G (IgG)	1350 mg/dl	955 - 1995 mg/dl
Total immunoglobulin A (IgA)	250 mg/dl	85 - 214 mg/dl
Total immunoglobulin E (IgE)	395 IU/ml	11 - 210 IU/ml
Total immunoglobulin M (IgM)	152 mg/dl	65 - 372 mg/dl
Complex factor 3 (C3)	120 mg/dl	90 - 180 mg/dl
Complex factor 4 (C4)	30 mg/dl	10 - 40 mg/dl
Rheumatoid factor (RF)	<20 IU/ml	0 - 30 IU/ml
C-reactive protein (CRP)	8 mg/L	1 - 10 mg/L
Erythrocyte sedimentation rate (ESR)	35 mm/hr	<10

**Table 2:** Autoantibodies laboratory results ANCA: antineutrophil cytoplasmic antibodies

Autoantibodies	Lab Assessment
Perinuclear ANCA (p-ANCA)	Negative
Cytoplasmic ANCA (c-ANCA)	Negative
Atypical ANCA	Positive

**Table 3:** Urine analysis results

Urine analysis	Values	Normal
pH	7	4.6-7.8
Specific gravity (SG)	1028	1005 - 1030
Protein	30 mg / dl	<100 mg/dl
Haemoglobin	0.1 mg / dl	<1 mg/dl
White blood cells	0-1 per high-power field (hpf)	< 5 per hpf
Erythrocytes	30-32 per hpf	< 3 per hpf

On the third day of hospitalization, a biopsy was obtained from a skin lesion on the calf, and the histopathological findings were consistent with established leukocytoclastic vasculitis. The biopsy revealed perivascular and mural inflammatory infiltration of the capillaries, composed mainly of polymorphonuclear cells, numerous eosinophils, a few lymphocytes, and many macrophages. Endothelial cell swelling, fibrinoid degeneration of the vascular wall, perivascular inflammatory infiltration, and thickening of the collagen bundles of the reticular dermis were also noted. According to the histopathologist, these findings are observed in cases of allergic purpura, autoimmune disease, or infection.

Throughout the hospitalization, the patient remained afebrile and in good general condition. A slight improvement in the skin lesions was observed during the hospital stay despite the absence of any treatment. She was discharged in good general condition with a possible diagnosis of Henoch-Schönlein purpura. She was advised to undergo urinalysis every 15 days to support confirmation of the diagnosis, as the clinical manifestation was not typical. In addition, she was advised to remain under close follow-up by her pediatrician and to monitor for any changes in clinical symptoms.

**Figure 3:**
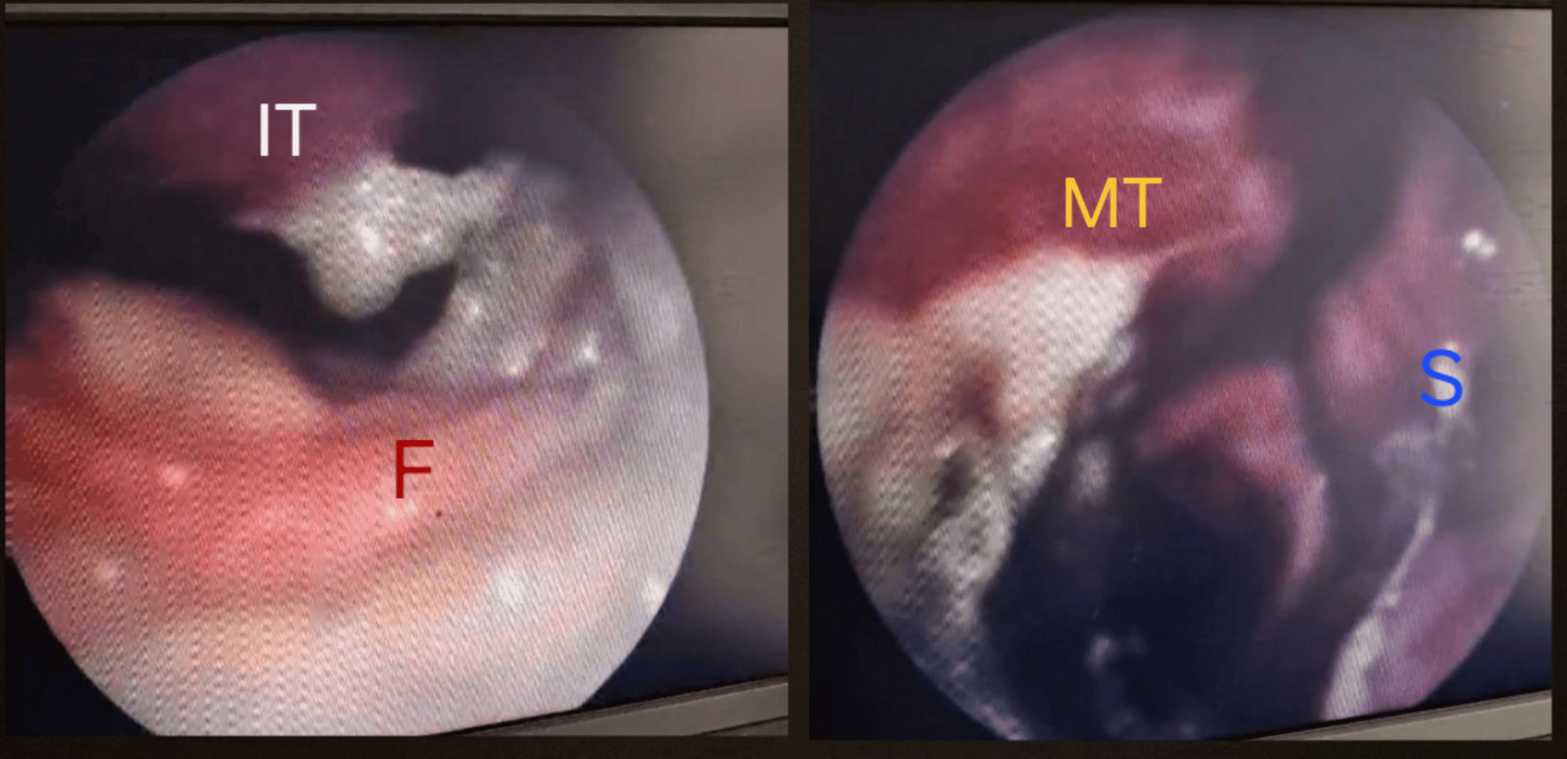
Endoscopic images of mucosal ulcers and crusts in the nasal cavity IT: inferior turbinate, F: floor of the nasal cavity, MT: middle turbinate, S: septum of the nose

**Figure 4:**
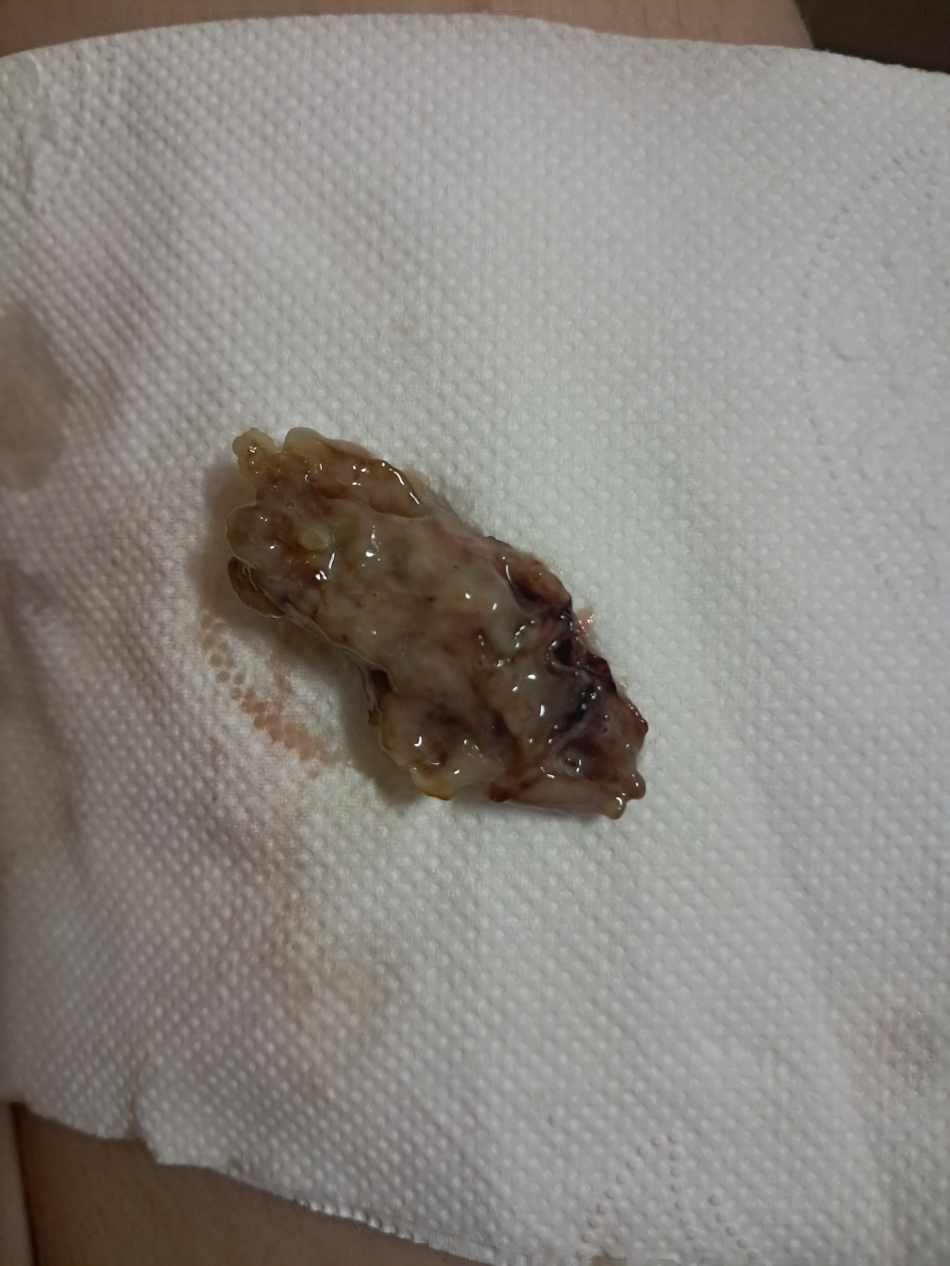
Nasal discharge, crust

A year later, the patient presented again with exacerbation of the haemorrhagic rash on both ankles, joint pain, and microscopic hematuria without proteinuria. The patient also complained of persistent nasal congestion, difficulty breathing through the nose, and episodes of epistaxis over the past year. She noted that the severity of her symptoms was correlated with menstruation or stress. She was admitted again to the hospital for further investigation. A new ENT evaluation was performed. The nasal endoscopy revealed extensive ulcerative lesions of the nasal mucosa and a severe degree of rhinitis with edema of the inferior and middle turbinates (Figure 3). Mucosal crusting and thick mucus nasal discharge were also observed (Figure 4). The following day, a biopsy of the nasal septal mucosa was obtained under local anaesthesia, which revealed a non-specific histopathological image of fibroblastic hyperplasia and was not diagnostic of any particular disease. The ENT specialist recommended continuation of topical nasal moisturizing ointment and nasal irrigation with saline solution (sodium chloride 0.9%). After one week, a second biopsy of the nasal mucosa was performed under local anesthesia, again without pathognomonic findings.

Furthermore, no specific genetic findings were identified from whole-exome sequencing. The patient remained in the hospital for observation, and she was discharged a few days later in improved condition without receiving any medication.

Three months later, due to intermittent microscopic hematuria, she underwent a kidney biopsy. The histopathological findings were compatible with IgAV nephritis, showing capillary and subendothelial IgA deposits and neutrophilic infiltration. She was started on corticosteroids, azathioprine, and hydroxychloroquine with subsequent improvement in nasal symptoms and joint pain. After 18 months of treatment, corticosteroids and hydroxychloroquine were discontinued. She is currently maintained on azathioprine. However, over the past six months, the patient has reported recurrence of symptoms, specifically joint pain and nasal congestion. Due to persistent nasal discomfort, a computed tomography (CT) scan of the facial bones was performed, which showed no abnormalities.

## Discussion

IgAV, also known as Henoch-Schönlein purpura, is the most common systemic vasculitis in children and typically presents with palpable purpura, arthralgia, abdominal pain, and renal involvement [7]. Although most pediatric cases follow a benign and self-limited course, atypical presentations can challenge early recognition and management, potentially leading to delayed diagnosis and prolonged monitoring [1, 8].

This case describes a 14-year-old girl with an atypical manifestation of IgAV, including persistent nasal mucosal ulcerations, recurrent vasculitic rash, and renal involvement with microscopic hematuria without proteinuria. Notably, nasal crusting and epistaxis are rarely reported in association with IgAV and are more frequently associated with other systemic ANCA-associated vasculitides [6, 9]. In particular, in 70-100% of patients with granulomatosis with polyangiitis (GPA), there is evidence of nasal and sinus problems [4, 10]. In our case, ANCA serology was non-specific, and histopathological examination of the nasal mucosa revealed non-diagnostic features, making GPA or other ANCA-associated vasculitis less likely. A study by Xiong et al. describes a correlation between IgAV and allergic rhinitis (AR) and/or chronic rhinosinusitis (CRS). In this study, patients with IgAV combined with AR or CRS accounted for 70.8% of the subjects, among whom IgAV combined with AR accounted for 56.9%, and IgAV combined with CRS accounted for 60.7%. The incidence of renal involvement and recurrent rash was significantly higher in the AR + CRS group than in the non-AR or non-CRS group [11].

The initial diagnosis was further complicated by the absence of classic systemic symptoms (such as abdominal pain or proteinuria) and the presence of psoriasiform skin lesions and oral ulcers. The presence of atypical ANCA and mildly elevated serum IgA levels added to the diagnostic uncertainty. The diagnosis was supported by a skin biopsy showing leukocytoclastic vasculitis and subsequently confirmed by a renal biopsy demonstrating IgA-dominant immune complex deposition consistent with IgAV nephritis. Several studies suggest that IgA immune complexes deposit in the mesangium and induce renal injury [12]. While IgA deposits in small vessels of different tissues, the nephritis is a common complication, so the renal biopsy is critical for the diagnosis and prognosis. The histological lesions of IgAV nephritis range from mild to severe, manifesting as slight mesangial proliferation, microscopic lesions, and focal nephritis in mild types, and diffuse proliferative nephritis, segmental glomerulosclerosis, or crescent formation in severe cases [13, 14]. Regarding nasal biopsy, granulomatosis with polyangiitis is histologically diagnosed on ENT biopsy specimens in nearly 80% of cases with ENT involvement [2]. However, the nasal biopsy, in order to diagnose IgAV, is not reported in the literature because nasal lesions have rarely been observed in this type of vasculitis.

In addition, our patient seemed to exhibit the symptoms longer than expected in pediatric patients, since in most cases the mean duration of symptoms is four weeks, after which they resolve automatically [15]. IgAV is considered a self-limited disease, but studies indicate that the disease course can be dynamic. In some cases, the disease can resolve without any significant renal involvement, while in others, the patient should be under close observation for renal complications [16]. In a study conducted by Villatoro-Villar et al. [12], patients were in complete response only 40% of the time, and relapses occurred in 10% of patients during the first five years of the disease.

This case underlines the diagnostic challenges posed by non-classical presentations of IgAV, especially in adolescents, where the disease may deviate from its typical pediatric course [8, 2]. According to the patient, nasal congestion and nasal crusting were the most distressing symptoms, significantly affecting her comfort and quality of life. The unusual ENT manifestations observed in this patient further expand the recognised clinical spectrum of IgAV and may reflect under-recognition of mucosal involvement. Our patient also demonstrated fluctuating symptoms that appeared to deteriorate with menstruation and emotional stress. This observation may need further exploration in pediatric populations with recurrent or stress-related relapse.

This case also emphasizes the importance of long-term multidisciplinary follow-up involving pediatrics, rheumatology, dermatology, nephrology, and otolaryngology to monitor disease progression and renal outcomes [17].

## Conclusions

IgAV is the most common cause of acute vasculitis in children; it is considerably less frequent in adults and is typically regarded as a self-limited condition. This case highlights the rhinological manifestations, which, together with arthralgia and cutaneous involvement, represented prominent clinical features in this patient. To the best of our knowledge, such an association has not been previously reported. This case contributes to expanding the spectrum of IgAV presentations and may support earlier recognition of atypical disease phenotypes. Non-typical presentations may result in diagnostic delay and postponement of appropriate specialist management, potentially increasing the burden on patients and caregivers. Therefore, a multidisciplinary approach and long-term follow-up are essential in the management of patients with atypical vasculitis.

## References

[1] Leung AK, Barankin B, Leong KF (2020). Henoch-Schönlein purpura in children: An updated review. Curr Pediatr Rev.

[2] Paulsen JI, Rudert H (2001). Manifestations of primary vasculitis in the ENT region [article in German]. Z Rheumatol.

[3] Marro J, Williams C, Pain CE, Oni L (2023). A case series on recurrent and persisting IgA vasculitis (Henoch Schonlein purpura) in children. Pediatr Rheumatol Online J.

[4] Comarmond C, Cacoub P (2014). Granulomatosis with polyangiitis (Wegener): clinical aspects and treatment. Autoimmun Rev.

[5] Cannady SB, Batra PS, Koening C, Lorenz RR, Citardi MJ, Langford C, Hoffman GS (2009). Sinonasal Wegener granulomatosis: a single-institution experience with 120 cases. Laryngoscope.

[6] Génin V, Rouau G, Agard C, Malard O, Néel A (2025). Ear, nose and throat manifestations in ANCA-associated vasculitis [article in French]. Rev Med Interne.

[7] Hastings MC, Rizk DV, Kiryluk K, Nelson R, Zahr RS, Novak J, Wyatt RJ (2022). IgA vasculitis with nephritis: update of pathogenesis with clinical implications. Pediatr Nephrol.

[8] Parums DV (2024). A Review of IgA vasculitis (Henoch-Schönlein Purpura) past, present, and future. Med Sci Monit.

[9] Bayindir Y, Jelusic M, Ozen S (2025). Highlights from the breakout session: vasculitis in paediatric rheumatology. Rheumatology (Oxford).

[10] Borner U, Landis BN, Banz Y, Villiger P, Ballinari P, Caversaccio M, Dubach P (2012). Diagnostic value of biopsies in identifying cytoplasmic antineutrophil cytoplasmic antibody-negative localized Wegener's granulomatosis presenting primarily with sinonasal disease. Am J Rhinol Allergy.

[11] Xiong W, Zhu Q, Hu X, Yuan Y, Zhao Y, Jing X, Guo Q (2024). Association of childhood IgA vasculitis with allergic rhinitis and chronic rhinosinusitis. Kidney Int Rep.

[12] Villatoro-Villar M, Crowson CS, Warrington KJ, Makol A, Ytterberg SR, Koster MJ (2019). Clinical characteristics of biopsy-proven IgA vasculitis in children and adults: a retrospective cohort study. Mayo Clin Proc.

[13] Xu L, Li Y, Wu X (2022). IgA vasculitis update: Epidemiology, pathogenesis, and biomarkers. Front Immunol.

[14] Luo F, Li Y, Zhang Y, Song Y, Diao J (2022). Bibliometric analysis of IgA vasculitis nephritis in children from 2000 to 2022. Front Public Health.

[15] Chen JY, Mao JH (2015). Henoch-Schönlein purpura nephritis in children: incidence, pathogenesis and management. World J Pediatr.

[16] Chatpaitoon B, Rianthavorn P, Chanakul A, Khaosut P (2024). Clinical characteristics and risk factors for kidney involvement in children with immunoglobulin A vasculitis. Pediatr Int.

[17] Wilkinson A (2019). Early recognition and treatment of Henoch-Schönlein purpura in children. Nurs Child Young People.

